# Ambrisentan use in a HIV-1 infected patient with end-stage renal disease and pulmonary hypertension: minimal removal by hemodialysis - a case report

**DOI:** 10.1186/s12882-019-1659-5

**Published:** 2020-01-28

**Authors:** José Ramón Santos, Ana Merino, Walter E. Haefeli, Cristina Miranda, Marisol Prats, Ioana Bancu, Lucía Bailón, José Moltó

**Affiliations:** 1 Fundacio Lluita contra la SIDA, Barcelona, Spain; 2 Infectious Diseases Department, Hospital Universitari Germans Trias i Pujol, Ctra de Canyet, s/n, 08916 Badalona, Barcelona, Spain; 3 Universitat Autònoma de Barcelona, Barcelona, Spain; 4 Nephrology Department, Hospital Universitari Germans Trias i Pujol, Barcelona, Spain; 5 Department of Clinical Pharmacology and Pharmacoepidemiology, Heidelberg University Hospital, Heidelberg, Germany; 6 Pulmonology Department, Hospital Universitari Germans Trias i Pujol, Barcelona, Spain

**Keywords:** Ambrisentan, Hemodialysis, Pulmonary arterial hypertension, HIV-infection, Drug interactions

## Abstract

**Background:**

Ambrisentan is a selective endothelin receptor antagonist used for the treatment of pulmonary arterial hypertension (PAH). Little is known about ambrisentan removal by hemodialysis in patients with end-stage renal disease (ESRD).

**Case presentation:**

A 53-year-old woman with HIV/hepatitis C virus (HCV) co-infection, PAH and ESRD on regular hemodialyis was admitted in our hospital due to refractory heart failure while on treatment with bosentan (125 mg twice daily) and tadalafil (20 mg once daily) for PAH and antiretroviral treatment (cART) including darunavir/cobicistat (800/150 mg once daily). Excessive exposure to bosentan due to drug interactions between bosentan and darunavir/cobicistat was suspected. Bosentan was replaced by ambrisentan, with progressive improvement in her clinical condition. Pre- and postdialyzer cocentrations of ambrisentan in plasma were determined and hemodialysis extraction ratio for ambrisentan was 2%.

**Conclusions:**

Our results suggest that hemodialysis results in minimal ambrisentan removal, and therefore no specific ambrisentan dosage adjustment seems to be required in ESRD patients undergoing hemodialysis.

## Background

The life expectancy of people living with HIV has shown significant improvement in recent decades, largely due to the widespread use of combined antiretroviral therapy (cART). However, this has been followed by an increase in the prevalence of non-AIDS-defining co-morbidities, including pulmonary arterial hypertension (PAH) and chronic kidney disease [1, 2]. The prevalence of PAH in the HIV population is approximately 0.5%, with a little variation since the introduction of cART. Nevertheless, the number of HIV-infected patients with end-stage renal disease (ESRD) requiring renal replacement therapy has increased.

Defining optimal dosing of many drugs in patients on hemodialysis may be difficult. Drug removal by hemodialysis is an essential factor in determining different dosing regimens. However, the pharmacokinetics of many drugs in patients on hemodialysis has been poorly studied, and information about their hemodialyzability remains scant [3–5]. In addition, HIV patients with PAH or ESRD are frequently affected by other co-morbidities that require specific therapies. These patients often receive many different drugs (polypharmacy), with a potential risk for drug-drug interactions among them [6, 7]. Here we report a clinical case involving an HIV-1-infected patient with severe PAH and ESRD on hemodialysis who was treated with ambrisentan with the intention to avoid drug interactions between bosentan and cART. Ambrisentan is a selective endothelin-A receptor antagonist, which is approved for the treatment of PAH [8, 9]. However, despite its better pharmacokinetic profile in comparison to bosentan, no data on ambrisentan pharmacokinetics in hemodialysis patients were available, so we evaluated the effect of hemodialysis on ambrisentan concentrations.

## Case presentation

The patient in this clinical case was a 53-year-old woman who was admitted to our hospital with severe respiratory failure. She had been diagnosed with HIV/hepatitis C virus (HCV) co-infection in 2000 as well as with PAH in 2002. In addition, she had ESRD and had been undergoing high-flux hemodialysis sessions since 2014 (vascular access: venous-arterial fistula; dialyzer: Revaclear® 400, capillary 101 dialyzer, membrane area 1.8 m^2^; Bicart® Gambro bicarbonate cartridge; blood flow: 350 mL/min; dialysate flow: 500 mL/min). The patient was undergoing 3 dialysis sessions per week and each session lasted approximately 4 h.

In January 2018, the patient was receiving cART with lopinavir/ritonavir (400/100 mg twice daily) plus raltegravir (400 mg twice daily), as well as bosentan (125 mg twice daily) plus tadalafil (20 mg once daily) for PAH. At that time, treatment with glecaprevir/pibrentasvir was indicated for HCV. To avoid drug interactions with HCV drugs, cART was switched to raltegravir (400 mg twice daily) plus rilpivirine (25 mg once daily), and bosentan and tadalafil were temporarily discontinued. In April 2018, after completing 8 weeks of HCV treatment, bosentan and tadalafil were reintroduced at the same doses as before HCV treatment, and rilpivirine was replaced by darunavir/cobicistat (800/150 mg once daily).

From April 2018 to September 2018 the patient had several episodes of congestive heart failure, which were satisfactory managed with medical treatment. Nevertheless, in September 2018 the patient presented with severe respiratory failure and clinical signs compatible with decompensated heart failure, and she had to be hospitalized. On her admission, the patient underwent daily hemodialysis sessions, and the negative fluid balance in each session was increased. However, the response to hemodialysis was poorer than expected, suggesting additional determinants of her clinical situation. In addition, during the admission, virologic failure was evidenced by an HIV-1 RNA load in plasma of 5424 copies/mL, with HIV evolution and development of new drug resistance mutations in the integrase gene (N155H) not present in previous genotypes. After ruling out other conditions, bosentan toxicity—probably because excessive exposure to bosentan due to drug interactions between bosentan and darunavir/cobicistat was considered. Bosentan was therefore replaced by ambrisentan with the intention of avoiding drug-drug interactions with cART. Additionally, cART was optimized, and the regimen was changed to darunavir/ritonavir (800/100 mg once daily) plus etravirine (200 mg twice daily) and dolutegravir (50 mg twice daily). Thereafter, the patient experienced progressive improvement in her clinical condition, HIV RNA in plasma was re-suppressed (< 40 copies/ml), and she was finally discharged to resume her usual hemodialysis program (3 sessions per week).

To evaluate the effect of hemodialysis on ambrisentan plasma concentrations, paired samples of blood entering (‘in’) and leaving (‘out’) the dialyzer and of the resulting dialysate were collected during one dialysis session. Blood samples were collected into heparin-lithium-containing 4-ml tubes 12.2 h after last ambrisentan dose. Plasma was isolated by centrifugation (3200 g for 15 min), and stored at − 80 °C until analysis. Dialysate (20 ml) was directly collected from the dialysate port of the dialyzer. Ambrisentan concentrations in both plasma and dialysate were determined using high-performance liquid chromatography coupled with tandem mass spectrometry (LC-MS/MS) using a validated method [10], and the hemodialysis extraction ratio (ER) for ambrisentan was calculated following standard procedures [5]. The patient gave and signed an informed consent before sampling took place.

The effect of hemodialysis on ambrisentan concentrations was evaluated during one hemodialysis session. Pre- and postdialyzer total ambrisentan concentrations were 42.8 ng/mL and 42.5 ng/mL, respectively, with negligible ambrisentan concentrations in dialysate (3.92 ng/mL). The hemodialysis extraction ratio was only 2.0%. At the end of the dialysis session (26.3 h after dosing), total ambrisentan concentrations in plasma were 35.6 ng/ml.

## Discussion and conclusions

The small differences between plasma ambrisentan concentrations going in and coming out of the dialyzer, together with barely noticeable ambrisentan concentrations in dialysate in this patient, suggest a minimal removal of ambrisentan by hemodialysis in patients with ESRD. This finding is consistent with the physiochemical characteristics of ambrisentan, which is 99% bound to plasma proteins, and is minimally eliminated by the kidneys, with only 3% of its dose being excreted unchanged into urine [8].

Besides the effect of hemodialysis on ambrisentan concentrations, the clinical case presented here highlights the complexity of drug-drug interactions affecting HIV-infected patients. In this case, mutual interactions between bosentan and cART were suspected. The patient had clinical signs of fluid retention associated with bosentan toxicity, which was presumably the result of excessive bosentan concentrations due to potent cytochrome P450 3A4 (CYP3A4) inhibition by cobicistat. Although no specific studies have evaluated the effect of cobicistat on bosentan pharmacokinetics, data with lopinavir/ritonavir have shown a rather substantial > 5-fold increase in bosentan exposure [11]. In the other direction, bosentan induces CYP3A4 activity [12], which is the main metabolic pathway for both darunavir and cobicistat. Previous studies have shown a decline in cobicistat exposure, and consequently inappropriately low darunavir concentrations, when darunavir/cobicistat is combined with the CYP3A4 inducers [13]. Consequently, combining bosentan with darunavir/cobicistat plus raltegravir could have resulted in functional monotherapy with raltegravir, leading to the virological failure and the development of new resistance mutations observed in this patient. Unfortunately, although it seems the most plausible hypothesis, no plasma samples to determine bosentan and antiretrovirals concentrations were available. Nevertheless, progressive improvement in the clinical condition of the patient along with re-suppression of HIV RNA after switching to ambrisentan and ART adjustment is concordant with our theory.

Ambrisentan is a selective endothelin-A receptor antagonist with proven efficacy and safety in the treatment of PAH [8]. In contrast to bosentan, ambrisentan is only a minor substrate of CYP3A4 and its elimination is mainly mediated by glucuronidation via several uridine-glucuronosyltransferase (UGT) isozymes [8]. Thus, the effect of potent CYP3A4 inhibitors on ambrisentan pharmacokinetics is much less compared to bosentan, and ambrisentan may be safely used in this scenario, as observed in our patient. Moreover, ambrisentan does not have any significant effect on the activity of UGT or cytochrome P450 isozymes or drug transporters, limiting the possibility of drug-drug interactions. It is noteworthy that ambrisentan concentrations at the end of the dialysis session were in the same range as those described in healthy volunteers receiving regular doses [14, 15], suggesting the absence of a need for dose adjustment in patients with ESRD.

Based on the minimal extraction ratio of ambrisentan by hemodialysis observed in our patient, no ambrisentan dose adjustment seems to be necessary in patients with PAH and ESRD undergoing hemodialysis. However, further investigations in this topic are recommendable. HIV-infected patients receiving polypharmacy may be affected by complex drug-drug interactions that require appropriate management, ideally by multidisciplinary teams with fluid communication among different prescribers.

## Data Availability

The datasets used and/or analysed during the current study are available from the corresponding author on reasonable request.

## Abbreviations

CART: Combined antiretroviral therapy
C_in_: Predialyzer ambrisentan concentration
C_out_: Postdialyzer ambrisentan concentration
CYP3A4: Cytochrome P450 3A4
ER: Extraction ratio
ESRD: End-stage renal disease
HCV: Hepatitis C virus
LC-MS/MS: Liquid chromatography coupled with tandem mass spectrometry
PAH: Pulmonary arterial hypertension
TP: Total protein
UGT: Uridine-glucuronosyltransferase
